# GDNF and miRNA-29a as biomarkers in the first episode of psychosis: uncovering associations with psychosocial factors

**DOI:** 10.3389/fpsyt.2024.1320650

**Published:** 2024-04-02

**Authors:** Marta Szwajca, Grzegorz Kazek, Natalia Śmierciak, Józef Mizera, Lucyna Pomierny-Chamiolo, Krzysztof Szwajca, Beata Biesaga, Maciej Pilecki

**Affiliations:** ^1^ Department of Psychiatry, Faculty of Medicine, Jagiellonian University Medical College, Kraków, Poland; ^2^ Department of Pharmacological Screening, Jagiellonian University Medical College, Krakow, Poland; ^3^ Department of Toxicology, Jagiellonian University Medical College, Kraków, Poland; ^4^ Faculty of Medicine and Health Sciences, Andrzej Frycz Modrzewski Krakow University, Krakow, Poland

**Keywords:** childhood trauma, GDNF, miRNA-29a, first episode of psychosis (FEP), premorbid adjustment, cognitive performance

## Abstract

**Aim:**

Schizophrenia involves complex interactions between biological and environmental factors, including childhood trauma, cognitive impairments, and premorbid adjustment. Predicting its severity and progression remains challenging. Biomarkers like glial cell line-derived neurotrophic factor (GDNF) and miRNA-29a may bridge biological and environmental aspects. The goal was to explore the connections between miRNAs and neural proteins and cognitive functioning, childhood trauma, and premorbid adjustment in the first episode of psychosis (FEP).

**Method:**

This study included 19 FEP patients who underwent clinical evaluation with: the Childhood Trauma Questionnaire (CTQ), the Premorbid Adjustment Scale (PAS), the Positive and Negative Syndrome Scale (PANSS), and the Montreal Cognitive Assessment Scale (MoCA). Multiplex assays for plasma proteins were conducted with Luminex xMAP technology. Additionally, miRNA levels were quantitatively determined through RNA extraction, cDNA synthesis, and RT-qPCR on a 7500 Fast Real-Time PCR System.

**Results:**

Among miRNAs, only miR-29a-3p exhibited a significant correlation with PAS-C scores (r = -0.513, p = 0.025) and cognitive improvement (r = -0.505, p = 0.033). Among the analyzed proteins, only GDNF showed correlations with MoCA scores at the baseline and after 3 months (r = 0.533, p = 0.0189 and r = 0.598, p = 0.007), cognitive improvement (r = 0.511, p = 0.025), and CTQ subtests. MIF concentrations correlated with the PAS-C subscale (r = -0.5670, p = 0.011).

**Conclusion:**

GDNF and miR-29a-3p are promising as biomarkers for understanding and addressing cognitive deficits in psychosis. This study links miRNA and MIF to premorbid adjustment and reveals GDNF’s unique role in connection with childhood trauma.

## 1 Introduction

Schizophrenia is a chronic and severe mental illness that has been ranked among the top 15 causes of disability worldwide (1). Most patients with schizophrenia experience multiple relapses, characterized by exacerbation of psychotic symptoms, leading to impaired social and occupational functioning, hospitalization, increased risk of suicide, and reduced quality of life (2). People suffering from schizophrenia are characterized by two or three times higher mortality compared to the general population (3) and shorter average life expectancy by 10-20 years (4, 5).

Proper and early diagnosis of schizophrenia is difficult due to the unclear pathophysiology and etiology of the disease, with diagnostic criteria based on subjective interpretation of symptoms. Treatment is largely empirical and experimental (6, 7). Furthermore, treatment failure is common in schizophrenia, with relapse rates of up to 80% within 5 years of the first episode (8).

There is, therefore, a need for a multidimensional assessment of schizophrenia risk factors in order to improve diagnosis, treatment, and prognosis. Schizophrenia appears to be a disorder with complex psycho-bio-social risk factors. After decades of searching for genes responsible for the development of schizophrenia, current research largely focuses on tracking the interactions between various and stage-specific risk factors of biological and environmental origin. Traditionally, significance has been attributed to the role of environmental factors (especially the history of childhood trauma), cognitive impairments, and premorbid social adjustment.

Exposure to childhood trauma is one of the extensively studied environmental factors in recent years and has been demonstrated to elevate the likelihood of developing psychosis (9, 10). 52-73% of patients with a first episode of psychosis (FEP) have a history of childhood trauma (11) and adverse childhood experiences raise the risk of psychosis by 2 to 4 times (12). Moreover, child abuse severely affects the course and outcome of psychosis, leading to functional and social impairment (13–17). It contributes to an increase in suicidal thoughts and attempts (18, 19), reduced response to antipsychotic treatment (20), lower rates of remission, extended hospital stays, a higher likelihood of treatment non-compliance and involuntary admissions (21–24), decreased engagement in rehabilitation (25), and exacerbation of psychotic symptoms (26, 27). Despite the significant role childhood trauma plays as a risk factor in the development and progression of psychosis, the specific mechanisms leading to the onset of psychosis remain unknown.

Furthermore, a decline in premorbid social functioning is a frequently observed characteristic of schizophrenia (28), and poor premorbid functioning is associated with an earlier age at psychosis onset, poorer overall clinical functioning, psychosocial and functional outcome, poorer treatment responsiveness, and poorer quality of life (29–32). Premorbid adjustment is an important predictor of conversion to psychosis (33–35).

In addition to the clinical symptoms of psychosis, most people with psychotic disorders also exhibit severely impaired cognitive functions that influence prognosis (36–38). Cognitive impairments are a pervasive feature in individuals experiencing the first episode of schizophrenia. These deficits are evident even prior to the onset of psychotic symptoms and persist throughout the entire course of the illness (38–40). Despite the availability of antipsychotic treatments, cognitive functioning often remains impaired in schizophrenic patients (40). Cognitive deficits are essential to consider as they have a significant impact on social and vocational functioning outcomes in patients with psychosis (41). The mechanisms underlying cognitive deficits are still not fully understood and are an area of ongoing research.

Despite identifying several prognostic factors in schizophrenia, predicting its risk, or assessing disease severity and progression remains challenging due to the complex nature of the disorder and the diversity of symptoms (42, 43). Therefore, there is an urgent need to find objective biomarkers that can facilitate early diagnosis and the assessment of disease progression in schizophrenia. The existing literature suggests that the disruption of glial cell line-derived neurotrophic factor (GDNF) and miRNA-29a is significant in schizophrenia (44–48).

GDNF is a glycosylated disulfide-bonded homodimer that is distantly related to the transforming growth factor-β superfamily (49). GDNF is the most well-known neurotrophic factor for dopaminergic neurons, promoting proliferation, differentiation, and protection of dopaminergic neurons as well as their survival and plasticity in the developing and adult brain (50). GDNF plays a crucial role in regulating dopamine transporters by promoting dopamine release (51) and turnover (52). Given the importance of dopamine neurotransmission changes in schizophrenia during adolescence and adulthood (53), research has focused on GDNF’s role in its pathophysiology. Studies have shown that an increase in endogenous GDNF in the brain induces molecular, cellular, and functional changes in dopamine signaling. This includes increased striatal presynaptic dopamine levels and reduced dopamine in the prefrontal cortex, resulting in dopaminergic abnormalities resembling those observed in schizophrenia (44). Additionally, an increase in endogenous GDNF leads to schizophrenia-like behavioral abnormalities in mice, such as apathy or avolition (44). However, studies monitoring peripheral levels of GDNF in schizophrenic subjects have yielded inconsistent results. Some studies found no significant differences in GDNF serum levels between patients with schizophrenia and healthy controls (54, 55), while others reported elevated GDNF levels in the cerebrospinal fluid of unmedicated FEP patients (44). Conversely, additional research demonstrated that baseline serum GDNF levels were significantly lower in drug-free schizophrenic patients (45) and chronically treated schizophrenic patients (46) compared to healthy controls. Consequently, the relationship between GDNF and schizophrenia remains unclear based on the above-mentioned results.

Small molecules known as microRNAs (miRNAs) have emerged as novel regulators in the pathophysiology of mental illnesses, including schizophrenia. These belong to a class of short, endogenously initiated non-coding RNAs that post-transcriptionally regulate gene expression through translational repression or mRNA degradation (56). Growing evidence suggests that miRNAs play a central role in regulating the development and function of the central nervous system (CNS), control dendrito- and synaptogenesis in early development and influence plasticity in the mature brain (57), ultimately affecting learning and memory (58). MicroRNA-29a plays a prominent role in the regulation of brain development and functioning due to its high expression in the CNS, particularly in hippocampal neurons, where its expression level is significantly higher than that of other members in the miRNA-29 family (59). MicroRNA-29a promotes proliferation (60), modulates neuronal survival (61), exerts a neuroprotective effect (62), promotes axon branching (59), and plays a key role in the regulation of serotonin 5-HT7R-dependent structural plasticity of the hippocampus (63), which is interesting in the context of hippocampal alterations in schizophrenia (64). There is some indication that miRNA-29a may be dysregulated in the brains of schizophrenic patients and potentially linked to neurological processes involved in this disorder (65). While research on the role of microRNA-29a in schizophrenia appears promising, there is a limited number of studies conducted on this subject and, to the authors’ knowledge, none have been conducted in the FEP group. One study demonstrated decreased levels of miRNA-29a in the prefrontal cortex of individuals with schizophrenia compared to healthy subjects (47). On the other hand, another study observed an upregulation of miRNA-29a in the peripheral blood of schizophrenic patients (48).

The aim of this study was to identify, using a hypothesis-free approach, the relationships between the expression of selected miRNAs and the levels of neural proteins in the plasma of patients with a first episode of psychosis in schizophrenia and cognitive functioning and its improvement during treatment. Additionally, we analyzed these biological parameters in the context of history of childhood trauma and premorbid adjustment.

## 2 Materials and methods

### 2.1 Study participants

This study included 19 patients, who were admitted to the inpatient wards of the Adult, Child and Adolescent Psychiatry Clinic at the University Hospital in Krakow, diagnosed with acute polymorphic psychotic disorders (F23) according to the International Statistical Classification of Diseases and Related Health Problems, 10th revision (ICD-10) (66). Moreover, all the subjects fulfilled the schizophrenia (F20) criteria as outlined in ICD-10, a diagnosis that was rigorously validated in a 3-month follow-up period. The diagnosis process was conducted by two independent experienced psychiatrists, who relied on the findings of clinical examinations performed upon admission to the ward and throughout the 3-month treatment period.

Participants gave informed written consent to take part in the study, and in the case of participants under 18 years of age, additional consent of their legal guardians was obtained. The participants could withdraw consent at any stage of the study. Patients were recruited after the approval of the study protocol issued by the Bioethics Committee of Jagiellonian University (KBET 122.6120.23.2016). The exclusion criteria included: inability to express individual consent, intellectual disability, occurrence of cardiovascular diseases, severe concomitant somatic disorders (e.g., diabetes mellitus, oncological or autoimmune diseases), history of CNS disorders (e.g., Alzheimer’s and Parkinson’s diseases), abuse of psychoactive substances or nicotine use within 3 months before the research, the occurrence of affective symptoms, and previous use of antipsychotic or antidepressant drugs.

### 2.2 Clinical evaluation

The clinical assessment, supported by supplementary psychiatric and psychological examinations, encompassed the utilization of various tools. These included the Childhood Trauma Questionnaire (CTQ), the Premorbid Adjustment Scale (PAS), the Positive and Negative Syndrome Scale (PANSS), and the Montreal Cognitive Assessment Scale (MoCA). The PANSS and MoCA assessments were conducted both prior to admission to the hospital and subsequently after the 12-week treatment period.

#### 2.2.1 CTQ

CTQ is a retrospective self-assessment tool providing a description of trauma experienced in childhood and adolescence (67, 68). It consists of 28 items, with each item rated on a 5-point scale with response options ranging from “never true” to “very often true”. CTQ measures five types of trauma: emotional abuse (CTQ-EA), physical abuse (CTQ-PA), sexual abuse (CTQ-SA), emotional neglect (CTQ-EN), and physical neglect (CTQ-PN), and gives the total result (CTQ-Total). The score for each type of abuse ranges from 5 to 25 points. Higher scores on each subscale suggest a higher severity of that specific type of trauma. A higher total score indicates a greater overall severity of trauma. CTQ can be considered as the most frequently used scale to assess child abuse in the literature (69). The Polish version of CTQ was used in this study (70). CTQ was administered to patients when they were clinically stabilized within 3 months of admission.

#### 2.2.2 PAS

PAS (71) is a widely used and useful measure of premorbid functioning in patients with psychosis (72). It assesses the extent to which a person has achieved certain developmental goals at various stages of life prior to the onset of psychotic symptoms. Thus, functioning is estimated in four age ranges, i.e., subscales: childhood (up to 11 years old) (PAS - childhood), early adolescence (12-15 years old) (PAS - early adolescence), late adolescence (16-18 years old) (PAS - late adolescence) and adulthood (19 years and above) (PAS - adulthood) in five main psychosocial domains: (1) sociability and withdrawal, (2) peer relationships, (3) scholastic performance, (4) adaptation to school, and (5) capacity to establish social and sexual relationships. Information on premorbid adaptation was collected from all available data sources: medical records, and was based on interviews with patients and family members. Considering that the study involved young patients with an average age of 18.4 ± 3.4 years, we decided to focus solely on the PAS - childhood and PAS - early adolescence subscales for our analysis, excluding scales that cover later periods in life. PAS also contains a general scale (PAS – general) consisting of 9 items evaluating factors such as the level of best functioning achieved by the individual, as well as items related to the level of energy, degree of interest in particular areas of life or independence. The total PAS score (PAS – total) is calculated by averaging the scores obtained in each of the developmental subscales and the PAS-general scale. Ratings on each subscale are expressed as decimal numbers ranging from 0.0 to 1.0, with lower numbers representing better adaptation and higher values suggesting poorer functioning.

#### 2.2.3 PANSS

PANSS (73) is a 30–item semi-structured interview for measuring the severity of psychotic and related symptoms. PANSS is the “gold standard” for measuring symptoms of schizophrenia (74). PANSS is divided into three scales: positive PANSS P (items P1 to P7), negative PANSS N (items N1 to N7) and general psychopathology PANSS G (items G1 to G16). The responses were scored on a 7-point scale where 1 indicates an absence of the pathology and 7 indicates the maximum severity of the symptom evaluated. The total score is the sum of all points, called PANSS total (PANSS T) with scoring that ranges from 30 to 210 points. The positive scale questions include assessments of delusions, hallucinations, excitement, suspiciousness/persecution, and grandiosity. The negative scale addresses blunted affect, emotional and social withdrawal, lack of spontaneity and flow of conversation. The general psychopathology subscale focuses on non-specific indicators of psychopathology, such as poor attention, depression, disorientation, poor impulse control, and feelings of guilt. PANSS was assessed at the baseline and 12 weeks later.

#### 2.2.4 MoCA

MoCA (75) is a tool for detecting cognitive dysfunctions that is widely used in various clinical conditions due to its high sensitivity and specificity in detecting cognitive impairment (75, 76). The scale consists of 12 items assessing different areas of cognitive functioning: attention and concentration, executive functions, memory, language, visuospatial abilities, conceptual thinking, calculation, and orientation to time and place. The total score ranges from 0 to 30, with higher scores indicating better cognitive functioning. MoCA identifies cognitive functions in the range of mild cognitive impairment with a score value of less than 26. It was found that MoCA is a useful and sensitive tool for monitoring cognitive functions in a group of patients with schizophrenia (77–80). Patients were examined with MoCA at the baseline and 12 weeks later. Due to the repetition of measurements, two equivalent versions of MoCA were used, in the Polish adaptation (81).

### 2.3 Blood collection

Blood samples were collected twice: at the beginning of the study and after a 3-month period. The samples were obtained in the morning (between 7 and 9 a.m.) following an 8-hour fasting period and overnight rest. Polypropylene tubes with ethylenediaminetetraacetic acid (EDTA) as an anticoagulant were used. Samples were processed within 4 hours of collection. Blood samples were centrifuged at 1,000 g and plasma samples were aliquoted into small portions to screw-cap cryogenic tubes and stored at -80°C until the assays. All samples were visually checked for bilirubinemia, hemolysis, lipemia, or turbidity and no samples were rejected for this reason.

### 2.4 Multiplex assay for selected plasma proteins

The selection of biological markers was based on a comprehensive review of the relevant literature pertaining to potential markers associated with schizophrenia. For neuroprotein determinations in plasma, we used the Neuroscience Human ProcartaPlex Panel provided by Thermo Fisher Scientific, which offers the broadest panel of neuroproteins commercially available: S100B, GFAP, GDNF, BDNF, NGF-beta, FGF-21, TDP-43, CNTF, NRGN, NCAM-1, MIF, KLK-6, NF-H, UCHL1, CHI3L1/YKL-40, Tau (Total), Tau [pT181], and amyloid beta 1-42. This multiplex assay is based on Luminex xMAP technology that allows for simultaneous quantitative determination of multiple analytes in a single, low volume sample with a much larger dynamic range than ELISA (82, 83).

Assays were conducted according to the manufacturer’s instructions. Vials of Standard Mix with premixed lyophilized proteins were centrifuged at 2,000 g for 10 seconds, and after that reconstituted with 50 µl of Universal Assay Buffer. Then vials was gently vortexed and centrifuged again and stored on ice for 10 minutes. Standard protein solutions were pooled and filled with up to 250 µl of Universal Assay Buffer. 4-fold serial dilutions were performed to generate 7-point standard curves with varying concentrations for each protein. Universal Assay Buffer itself was used as a background sample. Plasma samples stored until assaying at -80°C were thawed on ice and centrifuged at 10,000 g for 10 min for debris elimination. Samples were stored on ice and loaded onto assay plates within ∼30 minutes of thawing.

Paramagnetic bead washing with precipitation and separation steps was performed after 2 minutes of holding the plate on a 96-well microplate magnetic stand (Thermo Fisher Scientific). Next, Wash Buffer was discarded by quick inversion and the plate was gently blotted on laboratory absorbent paper. Then, the plate was removed from the magnetic separator. This procedure was repeated twice in each wash step.

Beads Mix was vortexed for 30 seconds before use and gently mixed during pipetting. 50 µl of Beads Mix was added to each well and washed with 150 µl of Wash Buffer. Next, 25 µl of Universal Assay Buffer was added to each well. To the wells prepared in this way, 25 µl of standards, background and serum samples were added in triplicates. The plates were sealed with AlumaSeal PCR grade foil (Sigma Aldrich) and shaken on an orbital shaker for 30 minutes at 500 rpm. After 12-14 hours of incubation at 4°C, the plates were shaken again and washing steps were performed as described above. 25 µl of Detection Antibody Mixture was added to each well and the sealed plates were shaken for 30 minutes at room temperature. Wash steps were repeated and 50 µl of Streptavidin-PE was added and the plate was sealed and shaken again. After beads washing, 120 µl of Reading Buffer was added to each assay well and sealed plates were shaken for 5 min at 800 rpm before the detection step.

Signal acquisition was performed using a Luminex 200 system (Luminex Corporation) with xPonent^®^ 3.1 software according to the parameters given in the assay protocol. Calibration and verification of signal reading were performed according to the operation manual.

### 2.5 MicroRNA expression analysis using qRT-PCR

Total RNA was isolated using the MagMAX mirVana Total RNA Isolation Kit (Thermo Fisher Scientific) based on superparamagnetic bead technologies to bind and purify nucleic acids. This kit has been recommended by the manufacturer to isolate small RNAs in plasma. Isolation was conducted according to the manufacturer’s instructions with manual separation on a magnetic stand. Total RNA containing miRNA was isolated from 100 µl of the plasma sample. Genomic DNA was enzymatically eliminated with TURBO DNase. The obtained RNA samples were stored at -20°C until cDNA synthesis but no longer than 2 weeks.

Expression analysis of miR let-7-5p, miR-16-5p, miR-21-5p, miR-26a-5p, miR-29a-3p, miR-142-3p, miR-145-5p, miR-146a-5p, and miR-181a-5p was conducted. These miRNAs were chosen based on their role in schizophrenia or importance in neurobiological processes related to the pathomechanisms of mental disorders. Quantitative assays of miRNA were performed with TaqMan Advanced miRNA technology and reagents provided by Thermo Fisher Scientific. This method allows the detection of multiple miRNA targets from a single sample with high sensitivity and specificity quantification using RT-qPCR (84).

Synthesis miRNA cDNA was performed using the TaqMan Advanced miRNA cDNA Synthesis Kit (Thermo Fisher Scientific). The poly(A) tailing, adaptor ligation, reverse transcription and miR-Amp pre-amplification steps were performed according to the manufacturer’s recommendations with 2 µl of plasma sample volume. The reaction product was aliquoted and stored at -80°C until analyzed, for up to 1 month.

The qRT-PCR reactions were performed using the 7500 Fast Real‐Time PCR System (Applied Biosystems) in 10 μl of reaction volume. 5 μl of TaqMan Fast Advanced Master Mix (Thermo Fisher Scientific), 0.5 μl of specific TaqMan Advanced Assay (probe and primer sets mix) and 4.5 μl of diluted cDNA were added to the well. Samples were analyzed in triplicates. Reactions were performed in fast mode in the thermal profile: polymerase activation at 95°C for 20 seconds and 45 cycles of denaturation at 95°C for 3 seconds and annealing at 60°C for 30 seconds.

The following TaqMan Advanced miRNA Assays (Thermo Fisher Scientific, catalog number A25576) were used: let-7-5p (478575_mir), miR-16-5p (477860_mir), miR-21-5p (477975_mir), miR-26a-5p (477995_mir), miR-29a-3p (478587_mir), miR-142-3p (477910_mir), miR-145-5p (477916_mir), miR-146a-5p (478399_mir) and miR-181a-5p (478399_mir).

The baseline, threshold and threshold cycle (Ct) were determined automatically using 7500 Software ver. 2.3 (Applied Biosystems). The expression levels of mRNA were normalized using miR-16-5p as an endogenous control for each sample, since it is widely used for plasma miRNA studies. The transcript levels were calculated based on threshold cycle (Ct) values (85).

Results from the RT-qPCR were expressed as ΔCt, calculated as the difference between the Ct values of miR-x and miR-16a-5p (ΔCt = miR-x Ct – miR-16a-5p Ct) for two time points: at the beginning and at the end of the study, denoted as ΔCt T1 and ΔCt T2, respectively. The changes in miRNA expression over the course of the study were quantified as the difference in expression between the end and the beginning of the study, represented as [ΔCt T2 – ΔCt T1].

### 2.6 Statistical analysis

Descriptive statistics were used to determine mean and median values of continuous variables and standard deviation from the mean (SD). The differences between means were analyzed by the T-student test (normal distribution) or Mann-Whitney test (non – normal distribution). The correlation between continuous variables was assessed by the Spearman coefficient. Associations between categorical variables were analyzed using the Pearson χ2 test. Two-sided p-values of < 0.05 were considered significant. All statistical analyses were carried out using Statistica v.13.3 software.

Graphs presenting the results of biomarker assays and clinical evaluations were generated using GraphPad Prism version 6.0 for Windows (GraphPad Software, Boston, MA, USA). Fitting curves were created using the smoothing spline method with a specified number of three knots.

## 3 Results

### 3.1 Sample characteristics

The study included 19 patients with FEP who were admitted to the inpatient wards at the Psychiatry Department and assessed at two time points (at the beginning of treatment and three months thereafter). In line with the ICD-10 criteria, all patients received a diagnosis of acute polymorphic psychotic disorder (F23). 12 weeks after the beginning of hospitalization, a diagnosis of schizophrenia was confirmed (F20 in accordance with ICD-10).

The study encompassed young patients with an average age of 18.4 ± 3.4 years. The duration of untreated psychosis (DUP), defined as the time from the onset of the first positive symptoms to the start of effective treatment in the course of the first psychotic episodes (86), ranged from a minimum of 3 days to a maximum of 56 days. The general characteristics of the sociodemographic and clinical data for the study sample are presented in **Table 1**.

**Table 1:** Sociodemographic and clinical characteristics of patients participating in the study.

Study participants	
N (%)	19 (100.0)
Sex	
Female N (%) Male N (%)	9 (47.4) 10 (52.6)
Age (years)	
Mean ± SD Range Median	18.4 ± 3.4 15 – 29 17
Duration of illness (days)	
Mean ± SD Range Median	60.1 ± 22.0 18.2 – 111.0 54.7
Duration of untreated psychosis (days)	
Mean ± SD Range Median	13.5 ± 12.3 3 – 56 7
Duration of hospitalization (days)	
Mean ± SD Range Median	49.5 ± 21.6 14 – 106 50

The subjects had not been treated with neuroleptics before admission to the hospital. At the beginning of hospitalization and qualification for the study, all patients received pharmacological treatment with typical and atypical neuroleptics in monotherapy: 8 patients received haloperidol, and 11 received atypical neuroleptics. Throughout the treatment period, pharmacotherapy was optimized, and by the third month of treatment, all patients were exclusively receiving atypical neuroleptics, with 13 patients in monotherapy and 6 patients in combination therapy. The average doses of the medications, converted to the equivalent of chlorpromazine (CPZ) (87), were similar to medians, measuring 47 in the first week and 412 in the third month of treatment. The therapeutic doses administered were well tolerated by all patients in the first and the second point of the study.

### 3.2 Clinical evaluations

The results of clinical assessment scales are presented in **Table 2**. CTQ and PAS were conducted when patients were clinically stabilized within 3 months of admission. PANSS and MoCA were assessed at two time points: at the beginning of hospitalization and subsequently after the 12-week treatment period.

**Table 2:** The results of clinical assessment scales collected at two time points: at the beginning of hospitalization (T1) and after the 12-week treatment period (T2).

	T1 Mean ± SD Range Median	T2 Mean ± SD Range Median	*p* value for differences between medians in T2-T1	Absolute level of change (T2 - T1) Mean ± SD Range Median	Relative level of change* Mean ± SD Range Median
PANSS					
TOTAL	94.5 ± 17.6 69 – 130 **90**	53.1 ± 12.1 39 – 90 **51**	0.0001	-43.9 ± 18.0 [-88] – [- 20] **-38**	-45 ± 11% [-69] – [-22]% **-43%**
P	24.1 ± 6.4 15 – 36 **22**	9.8 ± 2.1 7 – 16 **10**	0.0002	-14.1 ± 5.3 [-26] – [-5] **-12**	-58 ± 9% [-76] – [-33]% **-57%**
N	23.1 ± 5.3 12 – 31 **24**	13.5 ± 3.8 7 – 22 **13**	0.0002	-9.5 ± 5.5 [-19] – [-2] **-9**	-40 ± 17% [-63] – [-8]% **-44%**
G	48.4 ± 10.2 25 – 66 **47**	28.0 ± 4.7 21 – 42 **28**	0.0002	-20.6 ± 11.0 [-46] – [- 2] **-21**	-40 ± 15% [-69] – [-5]% **-43%**
MoCA					
MoCA	15.8 ± 3.6 9 – 21 **16**	20.6 ± 4.4 13 – 28 **22**	0.0001	4.7 ± 1.6 3 – 9 **5**	31 ± 11% 19 – 58% **27%**
CTQ					
TOTAL		55.0 ± 16.2 32 – 88 **53**			
EN		15.8 ± 4.6 8 – 24 **15**			
EA		13.4 ± 4.4 7 – 23 **13**			
SA		5.3 ± 0.9 (5 – 9) **5**			
PN		11.8 ± 4.7 5 – 21 **11**			
PA		8.7 ± 3.2 5 – 16 **8**			
PAS					
C		0.61 ± 0.16 0.17 – 0.83 **0.65**			
EA		0.55 ± 0.19 0.25 – 0.90 **0.53**			
G		0.6 ± 0.2 (0.2 – 0.8) **0.61**			
TOTAL		2.3 ± 0.8 (0.7 – 4.0) **2.23**			

*relative to the value at the beginning of treatment.

PANSS, Positive and Negative Syndrome Scale; P, positive scale; N, negative scale; G, general psychopathology scale; T, total scale; MoCA, Montreal Cognitive Assessment Scale; CTQ, Childhood Trauma Questionnaire; EN, emotional neglect; EA, emotional abuse; SA, sexual abuse; PN, physical neglect; PA, physical abuse; Total, total scale; PAS, Premorbid Adjustment Scale; C, childhood; EA, early adolescence; LA, late adolescence; A, adulthood; G, general; Total, total scale. The bold values represent the median.

During the course of treatment, significant improvement was observed in cognitive functioning and psychotic symptoms of the patients (**Table 2**). Drawing on research by Yang et al. (79), which identifies a MoCA cut-off below 23 as indicative of severe cognitive impairment in schizophrenia, our study found that all participants initially showed severe impairment. After three months of treatment, significantly lower values were observed in all PANSS subscales compared to their respective values during the first week after hospital admission. Statistically significant improvement in MoCA results, reflected in an increase in scores, compared to the baseline at admission, is also evident. Despite this progress, 10 patients continued to exhibit scores signaling severe impairment at this time. History of sexual abuse was indicated only by one patient, therefore we excluded this subscale from the correlation analyses.

### 3.3 Correlation between clinical assessment scales and expression of miRNAs

Due to the low quality of material isolated from one patient, analysis of miRNA concentration was performed for 18 patients included in the study. The plasma transcript levels of miR let-7-5p, miR-21-5p, miR-26a-5p, miR-142-3p, miR-145-5p, miR-146a-5p, and miR-181a-5p did not show statistically significant correlations with any of the clinical assessment scales or the degree of improvement during treatment (**Supplementary Table 2**). Among all the analyzed miRNAs, only miR-29a-3p exhibited a statistically significant correlation with certain clinical scales, specifically with the PAS subscale. Moreover, statistically significant correlations between the improvement of cognitive functions and changes in expression during the 3-month treatment were exclusively observed for miR-29a-3p.

In the group of 18 patients, the mean expression of miR-29a-3p (ΔCt) before and after 12 weeks of treatment were 5.7 ± 0.9 and 5.4 ± 1.6, respectively, and this difference was not statistically significant (p = 0.327). The difference in the average expression of miR-29a-3p in the study group at the beginning of the study and after 3 months of treatment (ΔCt T2 – ΔCt T1) was -0.33 Ct and was not statistically significant (**Supplementary Table 2**). Expression levels of miR-29a-3p (ΔCt) in T1, T2 and the mean level of change (ΔCt T2 – ΔCt T1) did not significantly correlate with sociodemographic or clinical characteristics of patients (data not shown).

No statistically significant correlations were observed between the expression level of miR-29a-3p and the MoCA scale values at the beginning of the study and after 3 months of treatment. The relationship between the two parameters concerned only the level of change during the study.

**Figure 1:**
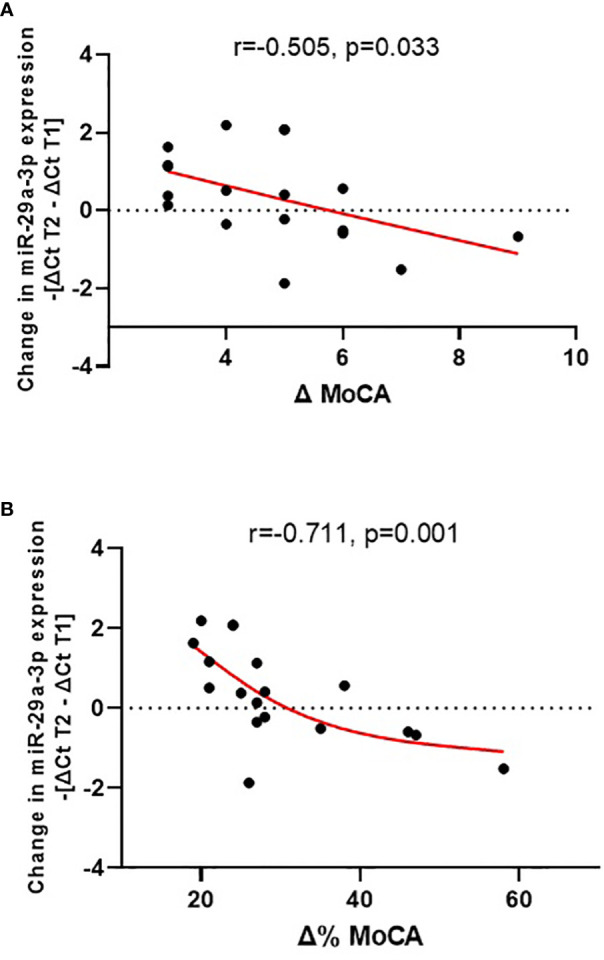
Change in plasma miR-29a-3p expression during treatment (-[ΔCt T2 – ΔCt T1]) vs. absolute cognitive improvement [Δ MoCA = MoCA T2 - MoCA T1; **(A)**] and vs. relative cognitive improvement [Δ% MoCA = 1 - MoCA T2/MoCA T1; **(B)**].

The change in plasma miR-29a-3p expression during the 3-month treatment correlated with an improvement in cognitive functioning, as indicated by an increase in MoCA scores (r = -0.505, p = 0.033), and with the relative degree of improvement compared to the value at the beginning of treatment (Δ% MoCA; r = -0.711, p = 0.001) (see **Figure 1**). In this case, the correlation is more pronounced and demonstrates a higher level of significance. Here, ‘absolute change’ refers to the straightforward difference in MoCA scores, while ‘relative change’ considers the percentage improvement relative to the initial score, providing a deeper understanding of patient progress.

All patients demonstrated cognitive improvement, however, changes in miR-29a-3p expression varied among the group. Specifically, 11 patients exhibited an increase in expression (mean 1.0 ± 0.7 Ct), while a decrease was observed in 7 patients (mean 0.8 ± 0.6 Ct).

Therefore, in further analyses, we decided to divide the patients into subgroups with increases (n=11) and decreases (n=7) in miR-29a-3p expression and performed a two-way frequency table analysis (**Table 3**). Based on the median degree of cognitive function improvement (5 points on the MoCA scale), patients had less (≤5) or greater (>5) cognitive function improvement. Of the 11 patients who had an increase in miR-29a-3p expression, 10 patients achieved worse improvement of cognitive functions, and only in one patient with an increase in the expression of this miRNA was a better improvement of cognitive functions observed.

**Table 3:** Two-way frequency table analysis in assessing the correlation between the direction of changes in miR-29a-3p expression and the degree of improvement in the MoCA scale.

	Patients with increased miR-29a-3p expression N (%)	Patients with decreased miR-29a-3p expression N (%)	*p* value χ2
Patients with improvement in MoCA score ≤5*	10 (76.9)	3 (23.1)	0.04
Patients with improvement in MoCA score >5*	1 (20.0)	4 (80.0)	

*median value.

**Figure 2:**
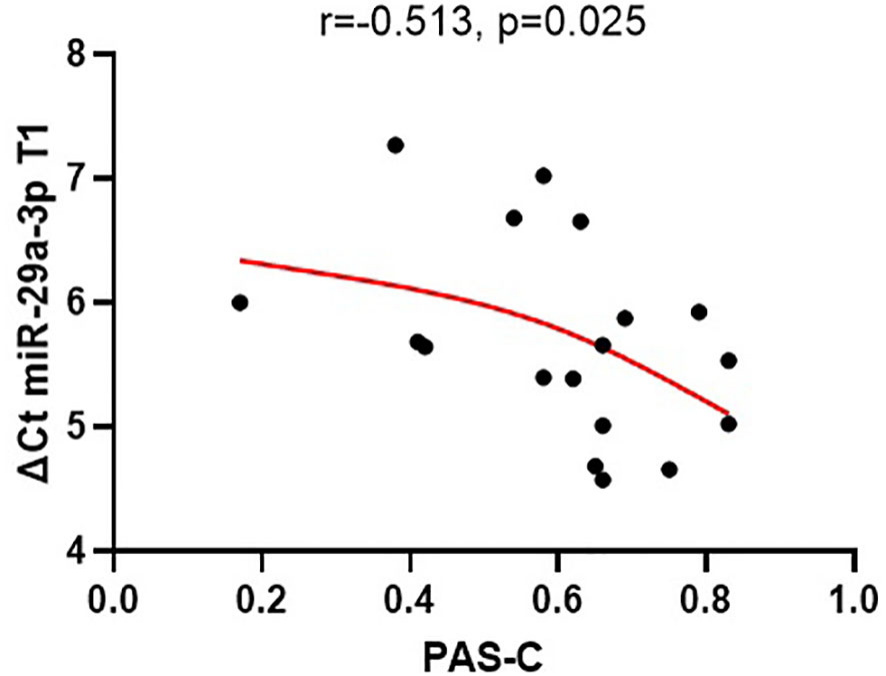
The relationships between the expression of miR-29a-3p (ΔCt) in plasma at the beginning of the treatment (T1) and PAS-C scores (premorbid adjustment during childhood).

The expression of miR-29a-3p in plasma exhibited significant correlation with PAS-C scores, indicating that elevated expression of miRNA at the beginning of hospitalization was associated with poorer premorbid adjustment during childhood (r = -0.513, p = 0.025) (see **Figure 2**). None of the other tested miRNAs showed any correlation with the analyzed PAS scales. (**Supplementary Table 3**).

### 3.4 Correlation between clinical assessment scales and concentration of the proteins

At the beginning of hospitalization, the level of 18 proteins derived from glial cells and neurons was determined in the plasma of study participants: amyloid beta 1-42, BDNF, CNTF, FGF-21, GDNF,GFAP, KLK-6, MIF, NCAM-1, NF-H, NGF -β, NRGN, S100B, Tau (total), Tau [pT181], TDP-43, UCHL1, and YKL-40.

**Figure 3:**
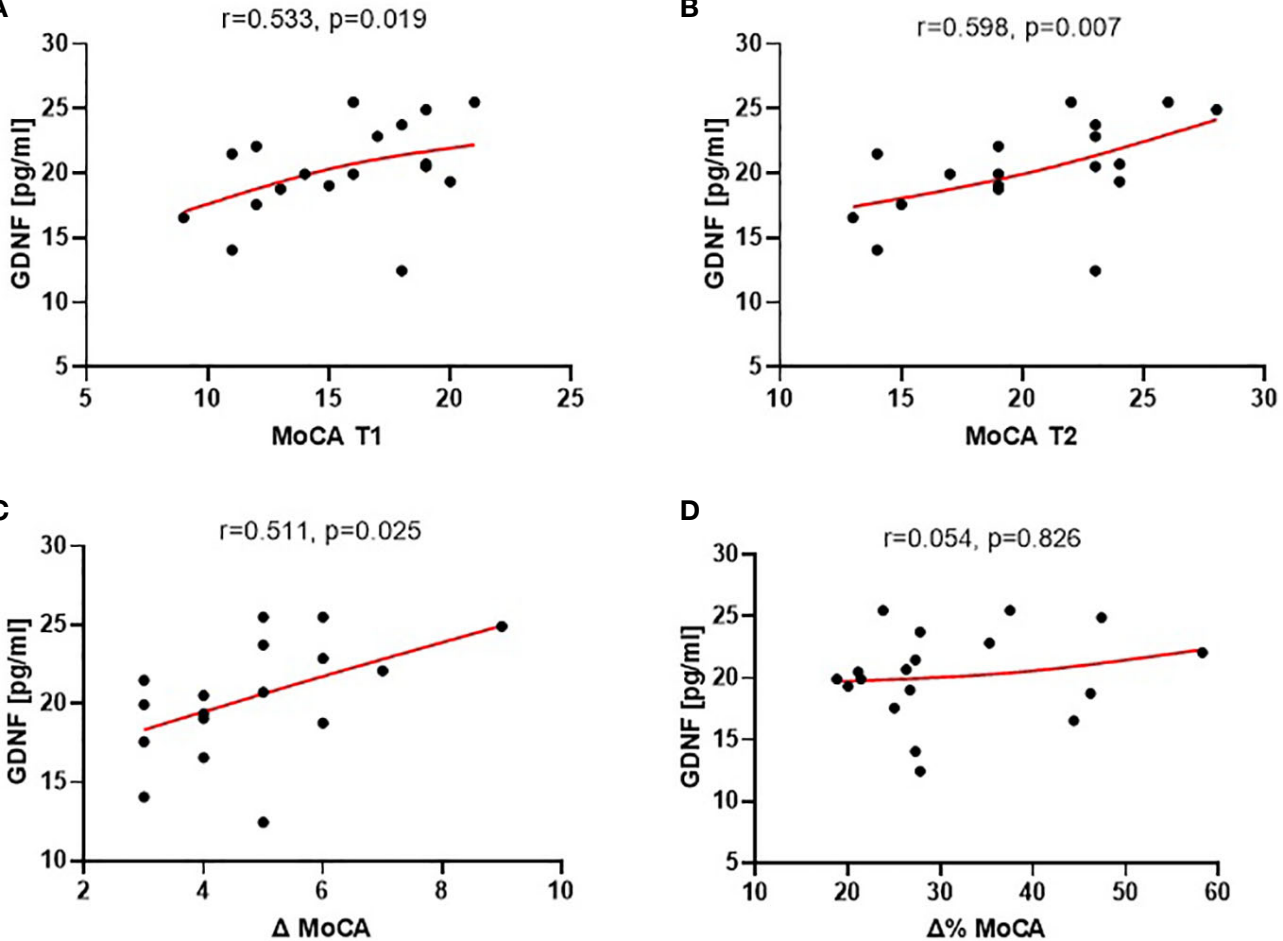
Correlations of plasma GDNF at the baseline (T1) with: MoCA scores at the baseline [T1, **(A)**], at end of study [T2, **(B)**], absolute cognitive improvement [Δ MoCA = MoCA T2 - MoCA T1; **(C)**], and relative cognitive improvement [Δ% MoCA = 1 - MoCA T2/MoCA T1; **(D)**] over the 3-month treatment period.

The analysis of the correlation between the concentration of these proteins and the MoCA scores revealed statistically significant correlations only with the level of GDNF (mean level 21.8 pg/ml ± 8.3) (**Supplementary Table 1**). Plasma GDNF concentration exhibited a positive correlation with MoCA scores both at the baseline and after 3 months of treatment (r=0.533, p=0.0189 and r=0.598, p=0.007, respectively) (as depicted in **Figure 3**). Furthermore, baseline GDNF showed a positive correlation with cognitive improvement observed over the 3-month treatment period, measured as the absolute change (increase) in MoCA scores (r=0.511, p=0.025).

**Figure 4:**
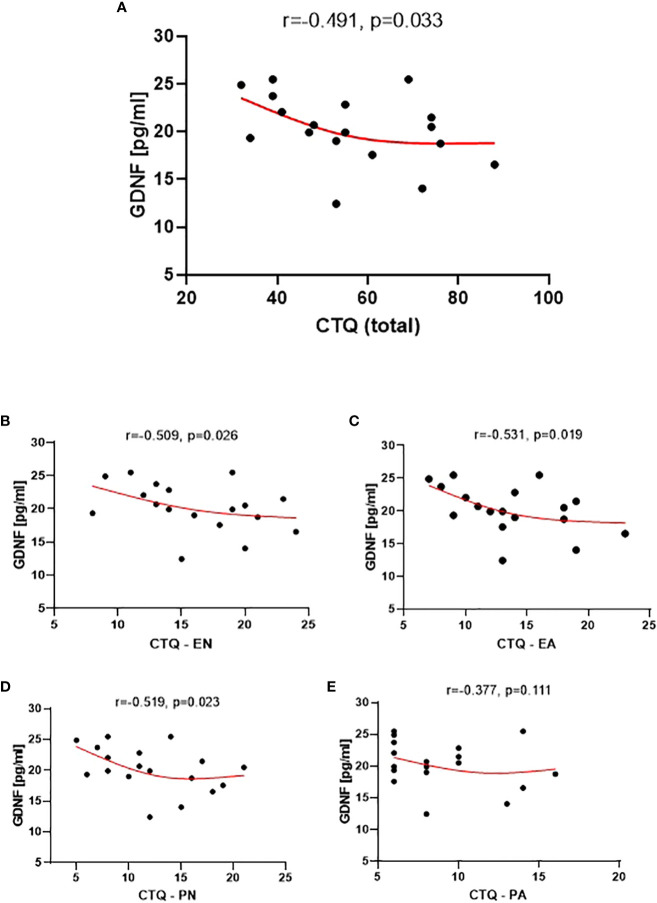
Correlation between GDNF level and the CTQ Total **(A)**, the CTQ EN subscale (Emotional Neglect) **(B)**, CTQ EA subscale (Emotional Abuse) **(C)**, CTQ PN subscale (Physical Neglect) **(D)**, and CTQ PA subscale (Physical Abuse) **(E)**.

The analysis of the correlation between the CTQ scores and the plasma levels of the tested neural proteins revealed a statistically significant correlation, which was only observed with GDNF (**Figure 4**). Specifically, lower levels of childhood traumatic experiences were associated with higher plasma levels of GDNF.

The GDNF level also showed significant correlations with the EN, PN, and EA subscales of CTQ (**Figure 4**). However, no statistical significance was observed for the correlation between the GDNF level and the PA subscale.

**Figure 5:**
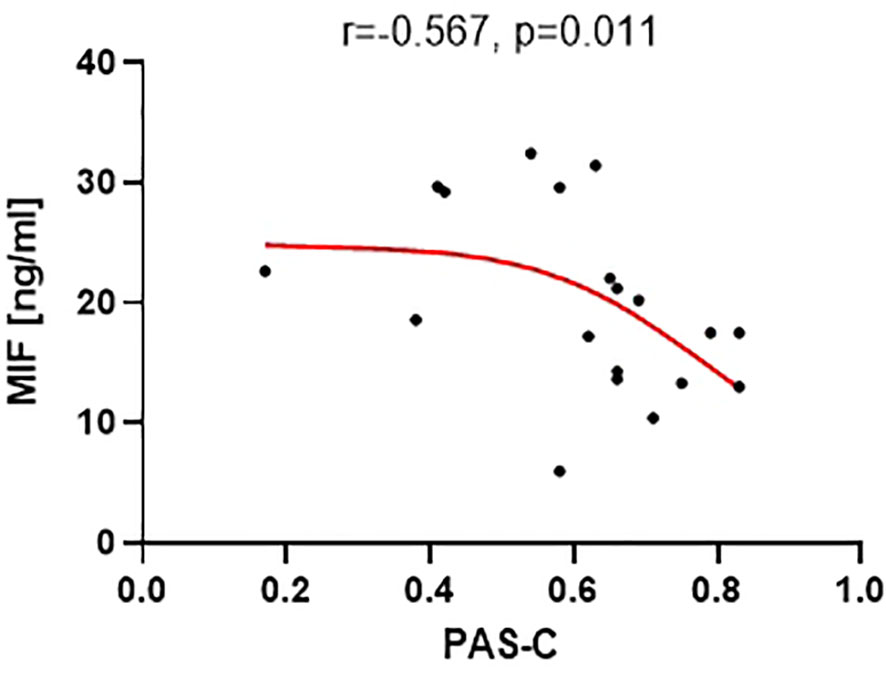
Correlation between MIF level and the PAS-C subscale (premorbid adjustment during childhood).

Out of the 18 analyzed neural proteins, only the concentration of macrophage migration inhibitory factor (MIF) (mean level 19.9 ng/ml ± 7.6) showed a significant correlation with the PAS-C subscale (r=-0.5670, p=0.011) (**Figure 5**). This correlation indicates that a higher level of MIF was associated with better premorbid functioning during childhood. However, the concentrations of the other proteins did not demonstrate significant correlations with the analyzed PAS scales.

## 4 Discussion

The findings of the present study showed that peripheral blood GDNF concentrations are closely related to cognitive functioning in individuals with first-episode psychosis. Higher levels of GDNF are associated with better cognitive performance both upon admission to hospital and at the three-month follow-up mark. To the best of our knowledge, this is the first study that has demonstrated that peripheral blood GDNF concentrations showed a positive correlation with cognitive improvement observed over the 3-month treatment period in FEP.

These findings suggest that GDNF may have a neuroprotective role in patients with FEP. The role of GDNF as a potent neurotrophic factor for midbrain dopamine neurons (88) that promotes striatal dopamine release and re-uptake (89) is particularly important given that cognitive dysfunctions in schizophrenia have been linked to aberrant dopaminergic transmission in the striatum (90, 91).

Our current finding of an association between GDNF protein levels and cognitive performance in psychosis is consistent with previous studies indicating that lower serum GDNF levels are associated with more severe cognitive deficits in first-episode schizophrenic patients and in those identified with the deficit subtype of schizophrenia (92, 93). Furthermore, our research complements these findings by showing that GDNF levels also serve as a predictor of cognitive improvement during an acute psychotic episode, especially during the course of treatment.

There is also a growing body of literature indicating the involvement of GDNF in neuronal plasticity. Heterozygous mutant mice with a targeted deletion of the *GDNF* gene have revealed impaired water-maze learning performance, indicating that GDNF is involved in hippocampal cognitive abilities (94). GDNF, acting through its GFRa1 receptor, appears to plays a crucial role in dendritic growth and synapse formation in hippocampal pyramidal neurons during early postnatal development (95). Its ability to control dendritic structure and spine density of adult-born hippocampal neurons is also thought to play an important role in regulating cognitive functions. The structural plasticity induced by GDNF may contribute to learning and memory processes by altering the connectivity and activity of hippocampal circuits (96), which is interesting because there is evidence of a reduction in adult-born hippocampal granule cell neurons in schizophrenia (97).

While the evidence suggests that GDNF may have a role in shaping neuronal structure and function, potentially contributing to the development and maintenance of cognitive abilities, the mechanisms underlying its effect on neuronal plasticity in the context of schizophrenia are still not fully understood. Given the potential neuroprotective role of GDNF and its association with cognitive functioning in individuals with first-episode psychosis, further research is warranted to elucidate the underlying mechanisms of its effects on neuronal plasticity and cognitive performance. Understanding the mechanisms by which GDNF modulates neuronal functioning and protects against cognitive decline may provide insights into the pathophysiology of psychosis and lead to the development of novel therapeutic strategies for cognitive disorders.

To the best of our knowledge, we are the first to demonstrate a significant correlation between improved cognitive functioning and the absolute change in miR-29a-3p expression in individuals experiencing their first episode of psychosis. This suggests the potential role of miR-29a-3p in the cognitive impairment associated with psychosis and highlights the importance of further investigating the underlying molecular mechanisms involved in this process.

MicroRNA-29a is crucial in regulating a variety of biological processes, such as neural proliferation, differentiation, survival, as well as influencing dendritic spine morphogenesis, neurite growth, axon branching, and inhibiting neuronal apoptosis (59, 61, 98–100). It promotes the proliferation of neural stem/progenitor cells, generating neurons, astrocytes, and oligodendrocytes in both developing and adult CNS (60). It also controls the levels of several plasticity-related epigenetic enzymes, and plays a critical role in the maturation of perineuronal nets (PNNs) (101). This is particularly interesting because alterations in PNNs have been suggested to be a key component of the pathophysiology of schizophrenia (102). MicroRNA-29a is also implicated in regulating structural plasticity dependent on 5-HT7R and the formation of hippocampal neural circuits during developmental processes (63). The role of miRNA-29a as a physiological modulator of 5-HT7R expression is intriguing, given the significant involvement of 5-HT receptors in cognitive impairments observed in psychotic disorders (103). Therefore, it seems that miRNA-29a is instrumental in neuronal survival and modulates neuronal cytoarchitecture and plasticity, which are crucial for cognitive functions. This highlights its significant impact in neurobiological processes relevant to cognitive health and disorders.

Overall, our study results indicate that the evaluation of GDNF and miR-29a-3p levels may be a promising approach for assessing cognitive status in patients with psychosis. The findings are consistent with previous research indicating that GDNF and miRNA-29a may be involved in neuronal plasticity, suggesting that further research on their effects on neuronal functioning may lead to the development of novel therapeutic strategies for cognitive disorders in psychosis.

Interestingly, miR-29a-3p may not only serve as a diagnostic biomarker for cognitive status in patients with psychosis but also potentially function as a significant indicator of premorbid functioning. It appears that we are the first to demonstrate the association between miR-29a-3p and premorbid adjustment. Our research has revealed that miR-29a-3p correlates with premorbid adjustment during childhood. Specifically, the higher expression of miR-29a-3p is associated with poorer adjustment during childhood in terms of sociability, withdrawal, or school adaptation. These additional findings further underscore the potential role of miR-29a-3p in the pathomechanism of psychosis. It is reasonable to suggest that miR-29a-3p could be a promising candidate for the prediction of illness course in schizophrenia and a valuable target for early intervention. Our findings align with previous research conducted in rodent models, indicating that social isolation can impact miRNA expression (104–108).

Notably, isolated rats consistently displayed altered levels of miR-29 family members compared to group-housed controls (108), highlighting that different social conditions can lead to specific miRNA changes. Moreover, studies have demonstrated that miRNA can modulate social behavior. For instance, overexpression of miRNA in the dentate gyrus of the hippocampus in adult rats subjected to neonatal isolation was shown to impact social behavior, regulating anxiety- and autistic-like behavior, spontaneous exploratory activities, and social engagement (105). In another study involving mice, miRNA changes resulted in stress-provoked aggressive behavior (106).

Dysregulation of miRNAs can significantly influence the ability to adapt behaviorally to social contexts, as seen in autism spectrum disorder (ASD) (109). Interestingly, miR-29a-3p is upregulated in children with ASD compared to controls, and a positive correlation was found between miR-29a-3p and autistic features (110), suggesting that miR-29a-3p plays a role in the development of social deficits.

We were the first to demonstrate the correlation indicating that a higher level of macrophage migration inhibitory factor was associated with better premorbid functioning during childhood. However, the precise mechanisms underlying this relationship are not entirely understood. Considering that MIF is a pleiotropic cytokine that plays a role in modulating both innate and adaptive immune responses, promoting the production or expression of a large panel of pro-inflammatory cytokines (111), elevated levels of MIF may be indicative of an enhanced immune response. This augmented immune activity has the potential to confer protection against specific neurological or psychiatric conditions, which, in turn, might contribute to enhanced premorbid adjustment. MIF is associated with the hypothalamic–pituitary–adrenal axis, a critical component of the stress response pathway (112, 113). MIF may impact the regulation of the HPA axis, which can be relevant to stress responses and the ability to adapt before the onset of illness. MIF has a significant role in promoting neurogenesis and neural protection by facilitating the proliferation and survival of neural cells through various signaling pathways (114, 115). This heightened neuroprotective activity of MIF may contribute to improved premorbid adjustment.

Our research emphasizes the distinctive role of GDNF. We have not only shown that peripheral blood GDNF concentrations are closely linked to cognitive functions in individuals experiencing their first episode of psychosis but also made the pioneering discovery that child abuse is associated with reduced GDNF levels in these patients. This further underscores the singular and pivotal role played by GDNF in this context. In recent years, there has been extensive research on the environmental factors that increase the likelihood of developing psychosis, and exposure to childhood trauma has been shown to be one of the most studied and significant contributors (9). Nonetheless, the specific mechanisms that explain the connection between childhood trauma and psychosis are not well understood. Our research findings suggest that child abuse may induce changes in GDNF expression among patients with psychosis. There are very few reports regarding the consequences of early life stress exposure on GDNF expression. In animal models, early life stress in the form of maternal separation leads to a 50% reduction in the concentration of GDNF in the cerebellum of mice (116). Furthermore, in the presence of 6-hydroxydopamine (6-OHDA), there were smaller increases in GDNF gene expression on rats exposed to early life stress in response to tissue injury compared to the normal control group. The results therefore indicate that early life adversity alone may cause changes that decrease the brain’s ability to protect itself against injury or insult (117). Moreover, in rats experiencing maternal deprivation in early life, up-regulation of DNA methylation at the GDNF gene promoter and the subsequent down-regulation of the GDNF gene expression in the ventral tegmental area (VTA) tissues were found. Maternal deprivation stress significantly reduced plasma dopamine and GDNF concentration (118). In the nucleus accumbens (NAc), the expression level of GDNF is significantly reduced in chronic early-life stressed mice, which are considered a stress-vulnerable strain (119). Our results are also consistent with studies among crack cocaine users. During the early stages of recovery from crack cocaine abuse, plasmatic GDNF was the factor that displayed changes associated with a history of childhood abuse (120). Taken together, this data suggests that prolonged alterations in GDNF expression may result from early-life stress exposure.

It has been proposed that an alteration in dopamine regulation as a result of adverse experiences in childhood plays a significant role in the development of psychosis (121). Exposure to adversity in childhood impacts the striatal dopamine function in adulthood in groups who are at ultra-high risk (UHR) of developing psychosis (122). Childhood trauma influences the relationship between ventral striatal dopamine release and induced positive psychotic symptoms (123). Decreased dopamine catabolism related to the COMT gene polymorphism increases psychosis proneness in individuals with a history of traumatic life events (124).

The function of GDNF as a regulator of the dopaminergic system is widely recognized. We hypothesize that the history of child trauma down-regulates the expression of GDNF and consequently weakens its ability to promote the survival and morphological differentiation of dopaminergic neurons (88, 125), promote neuronal survival from toxic damage (126), prevent apoptosis in the DA neuron population (127), protect DA neurons from oxidative stress (128), provide neurotrophic support to injured neurons in inflammatory conditions in CNS (129), its reparative effects on the nigrostriatal dopamine system (130, 131), promote dopamine synthesis, and enhance the capacity of augmented dopamine release and re-uptake (88, 89, 132). It could be assumed that child abuse may cause changes in GDNF expression which sensitizes the dopamine system, subsequently leading to the development of psychotic symptoms. However, the presented results do not provide direct evidence or investigate specific mechanisms, and further research is needed to explore these aspects. Overall, the study highlights the importance of understanding the mechanisms linking childhood trauma and psychosis, and the potential role of GDNF in this process.

## 5 Limitations

It is important to recognize several limitations in the present study. The cross-sectional nature of our data poses a limitation in establishing causal relationships between GDNF or miRNA levels and the functioning in individuals with first-episode psychosis. While our study has unveiled meaningful correlations, the lack of a longitudinal design prevents us from making definitive statements about causation or the dynamic changes over time. Another constraint that warrants consideration is the relatively small group size, which can be attributed to the exploratory nature of our research. Given the exploratory nature of our study, aimed at generating hypotheses for future research, we did not apply any correction for multiple comparisons (133, 134). Future studies with larger cohorts would enhance the robustness and generalizability of our results. Moreover, the absence of comparison groups, such as individuals with chronic schizophrenia, clinical high-risk populations, or healthy individuals, is a noteworthy limitation. While the absence of a control group was not imperative for fulfilling the study’s immediate objectives of identifying biological factors related to the functioning of patients with psychosis, it becomes crucial for future research to confirm and provide a more comprehensive understanding of the observed associations. Additionally, future studies should take into account other potential confounding factors, such as the co-occurrence of somatic diseases, the influence of pharmacotherapies, or a history of substance use.

## 6 Advantages

Our study group consisted mainly of young individuals, which resulted in relatively limited age diversity. One notable advantage of our study is the inclusion of patients experiencing their first episode of psychosis. This approach serves to minimize differences arising from disruptive factors like long-term pharmacotherapy or chronic disease processes. We employed a rarely-used approach in the field, considering both genetic markers in the form of miRNA and neuroproteomic markers. A significant strength of our study lies in the examination of multiple miRNAs and several neural-derived proteins. Crucially, we not only explored associations at the time of diagnosis but also identified the significance of miRNA in clinical improvement. Consequently, our study addresses the imperative need for research into the molecular mechanisms of schizophrenia, especially in cases where treatment effects prove inadequate. Furthermore, this research aligns with the current emphasis on the urgent development of biomarkers for schizophrenia.

## 7 Conclusions

The assessment of GDNF and miR-29a-3p levels presents a promising approach for evaluating cognitive status in individuals with psychosis. Elevated GDNF levels are notably correlated with improved cognitive performance, both upon initial assessment during hospital admission and throughout the three-month follow-up period. Importantly, our study is the first to establish a positive association between peripheral blood GDNF concentrations and cognitive enhancement observed over the three-month treatment period in individuals experiencing their first episode of psychosis. Additionally, we have unveiled a significant correlation between enhanced cognitive functioning and the absolute change in miR-29a-3p levels in first-episode psychosis patients. These findings suggest the potential utility of GDNF and miR-29a-3p as valuable biomarkers and contribute to a deeper understanding of their roles in addressing cognitive deficits in individuals with psychosis.

Our study is a pioneering report to elucidate the association of miRNA and MIF with premorbid adjustment during childhood. These findings reveal a distinct relationship between miR-29a-3p and MIF dysregulation and social adaptation, potentially underpinning the clinical manifestations of psychosis. These discoveries underscore the significant roles of miR-29a-3p and MIF in regulating social behavior and suggest that their dysregulation may influence neurobehavioral phenotypes, ultimately contributing to the development of social deficits in individuals with psychosis.

Lastly, our research accentuates the unique role of GDNF, not only in demonstrating a close association between peripheral blood GDNF concentrations and cognitive functions in individuals experiencing their first episode of psychosis, but also in pioneering the discovery of a connection between child abuse and reduced GDNF levels in such patients.

These collective findings suggest an important and noteworthy role for GDNF within this specific context.

The findings highlight the necessity for additional research aimed at comprehending the underlying mechanisms behind these discovered associations and exploring the potential of these molecules as biomarkers and therapeutic targets for cognitive disorders in individuals with psychosis. Moreover, the study emphasizes the significance of early intervention and prevention strategies for individuals exhibiting premorbid functioning decline and a history of childhood trauma.

## Data availability statement

The original contributions presented in the study are included in the article/**Supplementary Materials**, further inquiries can be directed to the corresponding author/s.

## Ethics statement

The studies involving humans were approved by the Bioethics Committee of the Jagiellonian University (KBET 122.6120.23.2016). The studies were conducted in accordance with the local legislation and institutional requirements. Written informed consent for participation in this study was provided by the participants as well as by their legal guardians.

## Author contributions

MS: Writing – review & editing, Writing – original draft, Methodology, Investigation, Data curation, Conceptualization. GK: Writing – review & editing, Writing – original draft, Methodology, Investigation, Conceptualization. NS: Writing – original draft, Methodology, Investigation, Data curation, Conceptualization. JM: Writing – original draft, Methodology. LP-C: Writing – review & editing, Writing – original draft. KS: Writing – review & editing, Writing – original draft, Investigation, Data curation. BB: Writing – review & editing, Writing – original draft, Methodology, Conceptualization. MP: Writing – review & editing, Writing – original draft, Supervision, Methodology, Investigation, Data curation, Conceptualization.
