# Supplementary material for: GDNF and miRNA-29a as biomarkers in the first episode of psychosis: uncovering associations with psychosocial factors
Source: Front Psychiatry. 2024 Apr 2;15:1320650. doi: 10.3389/fpsyt.2024.1320650 (PMC11027163; doi:10.3389/fpsyt.2024.1320650)
Supplement: Supplementary file 1 [file DataSheet_1.pdf]

SUPPLEMENTARY DATA

GDNF and miRNA-29a as Biomarkers in the First Episode of Psychosis: Uncovering Associations with Psychosocial Factors.

Table 1.

Spearman's rank correlation coefficient (above) and p-value (below), with values signifying p < 0.05 highlighted in red.

	KLK6 [ng/ml]	S100B [µg/ml]	UCHL1 [pg/ml]	GFAP [ng/m]l	NF-H [ng/ml]	YKL-40 /CHI3L1 [ng/ml]	CNTF [pg/ml]	Amyloid beta [pg/ml]	GDNF [pg/ml]	Tau [pg/ml]	p-Tau [pg/ml]	NCAM1 [ng/ml]	NGFβ [pg/ml]	BDNF [pg/ml	MIF [ng/ml]	TDP-43 [pg/ml]	NRGN ng/ml	FGF-21 [pg/ml]
**MoCA**																		
MoCA T1	-0.248	0.237	0.261	0.213	0.323	-0.013	-0.078	-0.079	**0.533**	-0.061	0.037	-0.4387	0.399	0.08	0.194	-0.012	-0.033	0.249
	0.303	0.329	0.281	0.381	0.177	0.957	0.75	0.748	**0.019**	0.805	0.88	0.134	0.09	0.744	0.426	0.963	0.892	0.303
MoCA T2	-0.043	-0.306	0.111	0.328	0.199	0.307	-0.102	-0.032	**0.598**	0.326	0.155	-0.063	-0.502	0.403	0.202	0.043	0.202	0.176
	0.86	0.203	0.652	0.17	0.413	0.201	0.683	0.897	**0.007**	0.173	0.526	0.797	0.028	0.087	0.407	0.866	0.407	0.472
MoCA Δ	-0.055	-0.363	-0.178	0.082	0.082	0.139	-0.183	0.112	**0.511**	0.088	0.361	-0.124	-0.191	0.288	0.055	0.281	0.056	0.006
	0.822	0.146	0.467	0.737	0.737	0.443	0.453	0.647	**0.027**	0.72	0.129	0.611	0.432	0.231	0.821	0.259	0.821	0.981
MoCA Δ%	-0.114	-0.372	0.169	-0.108	-0.096	-0.157	0.195	0.080	0.054	-0.076	-0.031	0.140	0.109	0.305	-0.004	0.157	-0.139	-0.339
	0.642	0.117	0.490	0.660	0.695	0.520	0.424	0.743	0.826	0.758	0.900	0.569	0.656	0.205	0.989	0.533	0.569	0.155
**CTQ**																		
EN	0.093	-0.308	0.245	-0.204	-0.353	0.081	0.028	-0.007	**-0.509**	-0.214	-0.0845	0.32	-0.383	-0.008	-0.155	-0.05	-0.159	-0.157
	0.704	0.199	0.312	0.403	0.138	0.742	0.909	0.979	**0.026**	0.379	0.731	0.182	0.106	0.973	0.527	0.842	0.515	0.521
EA	-0.116	-0.342	-0.235	-0.235	-0.327	-0.052	-0.117	-0.158	**-0.531**	-0.218	-0.12	0.252	-0.371	0.02	-0.213	0.041	-0.24	-0.305
	0.636	0.151	0.332	0.332	0.172	0.833	0.633	0.519	**0.019**	0.371	0.624	0.298	0.118	0.936	0.381	0.87	0.323	0.204
PN	-0.021	-0.344	-0.151	-0.071	-0.297	-0.009	0.049	-0.125	**-0.519**	-0.206	-0.034	0.116	-0.409	0.025	-0.197	0.004	-0.179	-0.034
	0.932	0.15	0.538	0.774	0.217	0.971	0.841	0.611	**0.023**	0.396	0.889	0.636	0.081	0.919	0.419	0.987	0.463	0.89
PA	-0.334	-0.382	-0.132	-0.19	-0.305	-0.034	-0.04	-0.092	-0.377	-0.165	-0.082	0.216	-0.283	0.077	-0.248	0.251	-0.37	-0.392
	0.162	0.136	0.591	0.435	0.204	0.889	0.871	0.708	0.111	0.5	0.74	0.373	0.24	0.754	0.306	0.314	0.118	0.097
TOTAL	-0.061	-0.186	-0.186	-0.194	-0.299	0.007	-0.027	-0.091	**-0.491**	-0.237	-0.088	0.286	-0.406	0.046	-0.186	0.048	-0.179	-0.132
	0.805	0.144	0.444	0.425	0.214	0.974	0.912	0.711	**0.033**	0.327	0.722	0.236	0.084	0.851	0.445	0.851	0.463	0.589
**PAS**																		
C	-0.118	-0.374	-0.188	0.321	-0.338	-0.12	-0.328	0.153	-0.294	0.082	0.083	-0.31	-0.305	-0.305	**-0.567**	0.149	-0.115	0.099
	0.631	0.114	0.44	0.18	0.157	0.626	0.171	0.532	0.224	0.739	0.735	0.498	0.205	0.205	**0.011**	0.544	0.639	0.686
EA	0.275	-0.406	-0.156	0.286	-0.098	-0.098	0.328	0.083	-0.224	0.037	-0.194	-0.276	-0.179	-0.179	-0.153	-0.205	-0.237	-0.056
	0.254	0.085	0.523	0.236	0.69	0.69	0.17	0.734	0.356	0.882	0.425	0.253	0.463	0.463	0.531	0.398	0.328	0.82
G	0.35	0.045	0.045	-0.117	0.291	0.291	0.261	0.329	-0.083	0.38	-0.024	0.341	-0.139	-0.139	-0.378	0.047	-0.198	-0.095
	0.141	0.853	0.853	0.633	0.226	0.226	0.281	0.169	0.735	0.109	0.921	0.154	0.57	0.57	0.111	0.85	0.416	0.699
TOTAL	-0.02	-0.007	-0.007	0.012	0.247	0.247	0.193	0.243	0.163	0.296	-0.203	0.088	0.01	0.01	0.007	0.088	-0.165	0.118
	0.935	0.976	0.976	0.96	0.309	0.309	0.428	0.317	0.504	0.219	0.404	0.721	0.969	0.968	0.977	0.721	0.499	0.631

Table 2.

Statistical parameters of miRNA expression at two time points: at the beginning of hospitalization (T1) and after the 12-week treatment period (T2).

	miRNA expression [ΔCt]						
	Before treatment (T1)		After treatment (T2)				
miR	X	SD	X	SD	*T*	*Z*	*p* value
miR let-7-5p	7.55	0.59	7.58	0.93	94	0.04	0.97
miR-21-5p	4.47	0.73	4.40	0.72	82	0.52	0.60
miR-26a-5p	5.28	1.58	5.25	1.57	63	0.26	0.80
miR-29a-3p	5.67	0.77	5.34	0.86	58	1.20	0.23
miR-142-3p	6.66	1.71	6.79	1.71	85	0.02	0.98
miR-145-5p	3.23	0.88	3.20	1.04	95	0.00	1.00
miR-146a-5p	3.59	0.83	3.16	0.99	70	1.01	0.31
miR-181a-5p	6.28	0.80	6.10	0.71	83	0.48	0.63

Table 3

Spearman's rank correlation coefficients for questionnaire scales versus miRNA expression, with p < 0.05 highlighted in red.

MoCA vs. miRNA ΔCt

	R	*p*
MoCA T1 & miR let-7-5p T1	-0.0203	0.9344
MoCA T1 & miR-21-5p T1	0.3981	0.0914
MoCA T1 & miR-26a-5p T1	0.2660	0.2711
MoCA T1 & miR-29a-3p T1	0.2994	0.2130
MoCA T1 & miR-142-3p T1	0.1673	0.4935
MoCA T1 & miR-145-5p T1	0.0229	0.9259
MoCA T1 & miR-146a-5p T1	0.1189	0.6278
MoCA T1 & miR-181a-5p T1	0.0845	0.7308

MoCA T2 & miR let-7-5p T2	0.1037	0.6727
MoCA T2 & miR-21-5p T2	0.4138	0.0782
MoCA T2 & miR-26a-5p T2	0.4048	0.1199
MoCA T2 & miR-29a-3p T2	-0.0146	0.9541
MoCA T2 & miR-142-3p T2	0.2078	0.4079
MoCA T2 & miR-145-5p T2	0.2056	0.3984
MoCA T2 & miR-146a-5p T2	0.2588	0.2848
MoCA T2 & miR-181a-5p T2	0.2605	0.2813

MoCA Δ & miR let-7-5p T1	-0.0701	0.7755
MoCA Δ & miR-21-5p T1	-0.1339	0.5847
MoCA Δ & miR-26a-5p T1	-0.3334	0.1630
MoCA Δ & miR-29a-3p T1	-0.2229	0.3590
MoCA Δ & miR-142-3p T1	-0.0144	0.9534
MoCA Δ & miR-145-5p T1	-0.1061	0.6657
MoCA Δ & miR-146a-5p T1	-0.1465	0.5495
MoCA Δ & miR-181a-5p T1	-0.2427	0.3168

MoCA Δ% & miR let-7-5p T1	-0.0439	0.8583
MoCA Δ% & miR-21-5p T1	-0.4032	0.0870
MoCA Δ% & miR-26a-5p T1	-0.3980	0.1300
MoCA Δ% & miR-29a-3p T1	-0.4032	0.0870
MoCA Δ% & miR-142-3p T1	-0.1054	0.6676
MoCA Δ% & miR-145-5p T1	-0.1862	0.4453
MoCA Δ% & miR-146a-5p T1	-0.1845	0.4497
MoCA Δ% & miR-181a-5p T1	-0.2863	0.2346

	R	*p*
MoCA Δ & miR let-7-5p T2-T1	-0.2346	0.3337
MoCA Δ & miR-21-5p T2-T1	-0.3415	0.1524
MoCA Δ & miR-26a-5p T2-T1	-0.0456	0.8668
**MoCA Δ & miR-29a-3p T2-T1**	**-0.5049**	**0.0326**
MoCA Δ & miR-142-3p T2-T1	0.1006	0.6913
MoCA Δ & miR-145-5p T2-T1	-0.3262	0.1728
MoCA Δ & miR-146a-5p T2-T1	-0.3775	0.1111
MoCA Δ & miR-181a-5p T2-T1	-0.3847	0.1039

MoCA Δ% & miR let-7-5p T2-T1/T1	0.0255	0.9176
MoCA Δ% & miR-21-5p T2-T1/T1	-0.2503	0.3013
MoCA Δ% & miR-26a-5p T2-T1/T1	0.1223	0.6518
**MoCA Δ% & miR-29a-3p T2-T1/T1**	**-0.7111**	**0.0009**
MoCA Δ% & miR-142-3p T2-T1/T1	0.1437	0.5695
MoCA Δ% & miR-145-5p T2-T1/T1	-0.2407	0.3209
MoCA Δ% & miR-146a-5p T2-T1/T1	-0.1941	0.4259
MoCA Δ% & miR-181a-5p T2-T1/T1	-0.3557	0.1350

PAS vs. miRNA ΔCt

	R	*p*
PAS EA & miR let-7-5p T1	0.3727	0.1160
PAS EA & miR-21-5p T1	-0.2497	0.3026

	R	*p*
PAS LA & miR let-7-5p T1	0.2072	0.3946
PAS LA & miR-21-5p T1	-0.0176	0.9429

PAS EA & miR-26a-5p T1	-0.1767	0.4693
**PAS EA & miR-29a-3p T1**	**-0.5134**	**0.0246**
PAS EA & miR-142-3p T1	0.1890	0.4384
PAS EA & miR-145-5p T1	-0.3323	0.1645
PAS EA & miR-146a-5p T1	0.0369	0.8807
PAS EA & miR-181a-5p T1	-0.1556	0.5247

PAS EA & miR let-7-5p T2	0.2585	0.2853
PAS EA & miR-21-5p T2	-0.1890	0.4384
PAS EA & miR-26a-5p T2	-0.4287	0.1352
PAS EA & miR-29a-3p T2	0.1914	0.4467
PAS EA & miR-142-3p T2	0.2328	0.3526
PAS EA & miR-145-5p T2	0.1556	0.5247
PAS EA & miR-146a-5p T2	0.0571	0.8163
PAS EA & miR-181a-5p T2	0.2664	0.2703

PAS EA & miR let-7-5p T2-T1	0.0149	0.9516
PAS EA & miR-21-5p T2-T1	-0.0870	0.7231
PAS EA & miR-26a-5p T2-T1	0.2371	0.3766
PAS EA & miR-29a-3p T2-T1	-0.4428	0.0657
PAS EA & miR-142-3p T2-T1	0.1231	0.6264
PAS EA & miR-145-5p T2-T1	-0.3552	0.1357
PAS EA & miR-146a-5p T2-T1	-0.0105	0.9658
PAS EA & miR-181a-5p T2-T1	-0.3886	0.1002

PAS LA & miR-26a-5p T1	-0.0476	0.8465
PAS LA & miR-29a-3p T1	-0.2116	0.3844
PAS LA & miR-142-3p T1	0.2937	0.2224
PAS LA & miR-145-5p T1	-0.2787	0.2480
PAS LA & miR-146a-5p T1	0.3748	0.1139
PAS LA & miR-181a-5p T1	0.0705	0.7741

PAS LA & miR let-7-5p T2	0.1631	0.5046
PAS LA & miR-21-5p T2	-0.2795	0.2464
PAS LA & miR-26a-5p T2	-0.4602	0.0729
PAS LA & miR-29a-3p T2	0.3280	0.1839
PAS LA & miR-142-3p T2	-0.1453	0.5651
PAS LA & miR-145-5p T2	-0.0882	0.7196
PAS LA & miR-146a-5p T2	0.0714	0.7714
PAS LA & miR-181a-5p T2	0.3466	0.1461

PAS LA & miR let-7-5p T2-T1	-0.1720	0.4815
PAS LA & miR-21-5p T2-T1	0.1464	0.5498
PAS LA & miR-26a-5p T2-T1	0.4425	0.0861
PAS LA & miR-29a-3p T2-T1	-0.3477	0.1574
PAS LA & miR-142-3p T2-T1	0.3622	0.1396
PAS LA & miR-145-5p T2-T1	-0.1517	0.5353
PAS LA & miR-146a-5p T2-T1	0.2108	0.3864
PAS LA & miR-181a-5p T2-T1	-0.2496	0.3028

CTQ vs. miRNA ΔCt	R	*p*
CTQ EN & miR let-7-5p T1	0.0062	0.9801
CTQ EN & miR-21-5p T1	-0.0923	0.7070
CTQ EN & miR-26a-5p T1	0.0264	0.9147
CTQ EN & miR-29a-3p T1	-0.2154	0.3759
CTQ EN & miR-142-3p T1	0.0097	0.9687
CTQ EN & miR-145-5p T1	-0.1090	0.6569
CTQ EN & miR-146a-5p T1	0.1978	0.4169
CTQ EN & miR-181a-5p T1	0.1407	0.5657

CTQ EN & miR let-7-5p T2	0.0255	0.9175
CTQ EN & miR-21-5p T2	-0.2453	0.3115
CTQ EN & miR-26a-5p T2	-0.2802	0.2931
CTQ EN & miR-29a-3p T2	0.0135	0.9578
CTQ EN & miR-142-3p T2	-0.1459	0.5635
CTQ EN & miR-145-5p T2	-0.2910	0.2268
CTQ EN & miR-146a-5p T2	-0.0774	0.7529
CTQ EN & miR-181a-5p T2	-0.0545	0.8246

CTQ EN & miR let-7-5p T2-T1	0.1468	0.5487
CTQ EN & miR-21-5p T2-T1	0.1916	0.4319
CTQ EN & miR-26a-5p T2-T1	0.2257	0.4007
CTQ EN & miR-29a-3p T2-T1	-0.0652	0.7972
CTQ EN & miR-142-3p T2-T1	0.1097	0.6649
CTQ EN & miR-145-5p T2-T1	0.1433	0.5584
CTQ EN & miR-146a-5p T2-T1	0.1820	0.4559
CTQ EN & miR-181a-5p T2-T1	0.1565	0.5223

CTQ EA & miR let-7-5p T1	0.0669	0.7854
CTQ EA & miR-21-5p T1	-0.2968	0.2172
CTQ EA & miR-26a-5p T1	-0.1427	0.5601
CTQ EA & miR-29a-3p T1	-0.3091	0.1978
CTQ EA & miR-142-3p T1	-0.1506	0.5383
CTQ EA & miR-145-5p T1	-0.1312	0.5923
CTQ EA & miR-146a-5p T1	-0.0026	0.9914
CTQ EA & miR-181a-5p T1	-0.0511	0.8355

CTQ EA & miR let-7-5p T2	0.0070	0.9772
CTQ EA & miR-21-5p T2	-0.2316	0.3400
CTQ EA & miR-26a-5p T2	-0.1596	0.5548
CTQ EA & miR-29a-3p T2	-0.0612	0.8095
CTQ EA & miR-142-3p T2	-0.1099	0.6642
CTQ EA & miR-145-5p T2	-0.1823	0.4551
CTQ EA & miR-146a-5p T2	-0.0493	0.8411
CTQ EA & miR-181a-5p T2	-0.0986	0.6879

CTQ EA & miR let-7-5p T2-T1	0.2052	0.3994
CTQ EA & miR-21-5p T2-T1	-0.0211	0.9316
CTQ EA & miR-26a-5p T2-T1	-0.0163	0.9523
CTQ EA & miR-29a-3p T2-T1	-0.0705	0.7810
CTQ EA & miR-142-3p T2-T1	-0.0612	0.8095
CTQ EA & miR-145-5p T2-T1	0.0643	0.7937
CTQ EA & miR-146a-5p T2-T1	0.0141	0.9543
CTQ EA & miR-181a-5p T2-T1	0.0018	0.9943

CTQ PN & miR let-7-5p T1	0.0862	0.7256
CTQ PN & miR-21-5p T1	-0.0950	0.6988
CTQ PN & miR-26a-5p T1	-0.0334	0.8919
CTQ PN & miR-29a-3p T1	-0.1971	0.4186
CTQ PN & miR-142-3p T1	0.0704	0.7746
CTQ PN & miR-145-5p T1	-0.0906	0.7121
CTQ PN & miR-146a-5p T1	0.2455	0.3110
CTQ PN & miR-181a-5p T1	0.1302	0.5952

	R	*p*
CTQ PN & miR let-7-5p T2	0.0396	0.8721
CTQ PN & miR-21-5p T2	-0.1989	0.4144
CTQ PN & miR-26a-5p T2	-0.2883	0.2790
CTQ PN & miR-29a-3p T2	0.0197	0.9382
CTQ PN & miR-142-3p T2	-0.1574	0.5327
CTQ PN & miR-145-5p T2	-0.3361	0.1594
CTQ PN & miR-146a-5p T2	-0.0220	0.9288
CTQ PN & miR-181a-5p T2	0.0642	0.7939

CTQ PN & miR let-7-5p T2-T1	0.1232	0.6154
CTQ PN & miR-21-5p T2-T1	0.1513	0.5363
CTQ PN & miR-26a-5p T2-T1	0.1582	0.5585
CTQ PN & miR-29a-3p T2-T1	-0.0829	0.7438
CTQ PN & miR-142-3p T2-T1	0.1668	0.5084
CTQ PN & miR-145-5p T2-T1	0.1452	0.5532
CTQ PN & miR-146a-5p T2-T1	0.1346	0.5827
CTQ PN & miR-181a-5p T2-T1	0.0607	0.8050

CTQ PA & miR let-7-5p T1	0.1377	0.5740
CTQ PA & miR-21-5p T1	-0.3741	0.1146
CTQ PA & miR-26a-5p T1	-0.2482	0.3056
CTQ PA & miR-29a-3p T1	-0.4448	0.0564
CTQ PA & miR-142-3p T1	-0.0625	0.7993
CTQ PA & miR-145-5p T1	-0.2926	0.2241
CTQ PA & miR-146a-5p T1	-0.1458	0.5513
CTQ PA & miR-181a-5p T1	-0.2074	0.3941

CTQ PA & miR let-7-5p T2	0.0426	0.8626
CTQ PA & miR-21-5p T2	-0.1812	0.4579
CTQ PA & miR-26a-5p T2	-0.0247	0.9278
CTQ PA & miR-29a-3p T2	0.0096	0.9697
CTQ PA & miR-142-3p T2	-0.0557	0.8262
CTQ PA & miR-145-5p T2	-0.0308	0.9004
CTQ PA & miR-146a-5p T2	0.0308	0.9004
CTQ PA & miR-181a-5p T2	-0.0553	0.8222

CTQ PA & miR let-7-5p T2-T1	0.1857	0.4466
CTQ PA & miR-21-5p T2-T1	-0.1667	0.4952
CTQ PA & miR-26a-5p T2-T1	-0.1495	0.5805
CTQ PA & miR-29a-3p T2-T1	-0.2496	0.3178
CTQ PA & miR-142-3p T2-T1	-0.0686	0.7869
CTQ PA & miR-145-5p T2-T1	-0.1495	0.5414
CTQ PA & miR-146a-5p T2-T1	-0.1621	0.5072
CTQ PA & miR-181a-5p T2-T1	-0.1341	0.5842

CTQ Total & miR let-7-5p T1	-0.0097	0.9687
CTQ Total & miR-21-5p T1	-0.1608	0.5108
CTQ Total & miR-26a-5p T1	-0.0905	0.7125
CTQ Total & miR-29a-3p T1	-0.2417	0.3189
CTQ Total & miR-142-3p T1	-0.0132	0.9573
CTQ Total & miR-145-5p T1	-0.0879	0.7206
CTQ Total & miR-146a-5p T1	0.1265	0.6057
CTQ Total & miR-181a-5p T1	0.0694	0.7776

CTQ Total & miR let-7-5p T2	0.0448	0.8554
CTQ Total & miR-21-5p T2	-0.2012	0.4087
CTQ Total & miR-26a-5p T2	-0.2504	0.3497
CTQ Total & miR-29a-3p T2	0.0662	0.7942
CTQ Total & miR-142-3p T2	-0.1169	0.6442
CTQ Total & miR-145-5p T2	-0.2311	0.3411
CTQ Total & miR-146a-5p T2	-0.0158	0.9488
CTQ Total & miR-181a-5p T2	-0.0114	0.9630

	R	*p*
CTQ Total & miR let-7-5p T2-T1	0.1169	0.6337
CTQ Total & miR-21-5p T2-T1	0.0940	0.7018
CTQ Total & miR-26a-5p T2-T1	0.1178	0.6639
CTQ Total & miR-29a-3p T2-T1	-0.1293	0.6092
CTQ Total & miR-142-3p T2-T1	0.0982	0.6981
CTQ Total & miR-145-5p T2-T1	0.1028	0.6753
CTQ Total & miR-146a-5p T2-T1	0.0571	0.8163
CTQ Total & miR-181a-5p T2-T1	0.0650	0.7914
